# Transglutaminase 2 as a Marker for Inflammation and Therapeutic Target in Sepsis

**DOI:** 10.3390/ijms22041897

**Published:** 2021-02-14

**Authors:** Ting Su, Xian-Yang Qin, Yutaka Furutani

**Affiliations:** 1 Liver Cancer Prevention Research Unit, RIKEN Cluster for Pioneering Research, Wako, Saitama 351-0198, Japan; ting.su@riken.jp (T.S.); xyqin@riken.jp (X.-Y.Q.); 2 Department of Intensive Care Unit, The Affiliated Drum Tower Hospital, Medical School of Nanjing University, Nanjing 210008, China

**Keywords:** transglutaminase, sepsis, antibacterial, antiviral, covalent crosslinking, inhibitor, Elafin

## Abstract

Sepsis results in lethal organ malfunction due to dysregulated host response to infection, which is a condition with increasing prevalence worldwide. Transglutaminase 2 (TG2) is a crosslinking enzyme that forms a covalent bond between lysine and glutamine. TG2 plays important roles in diverse cellular processes, including extracellular matrix stabilization, cytoskeletal function, cell motility, adhesion, signal transduction, apoptosis, and cell survival. We have shown that the co-culture of *Candida albicans* and hepatocytes activates and induces the translocation of TG2 into the nucleus. In addition, the expression and activation of TG2 in liver macrophages was dramatically induced in the lipopolysaccharide-injected and cecal ligation puncture-operated mouse models of sepsis. Based on these findings and recently published research, we have reviewed the current understanding of the relationship between TG2 and sepsis. Following the genetic and pharmacological inhibition of TG2, we also assessed the evidence regarding the use of TG2 as a potential marker and therapeutic target in inflammation and sepsis.

## 1. Introduction

Sepsis results in lethal organ dysfunction, which is increasing in prevalence due to the dysregulated host response to infection [1]. It is a global health crisis, affecting over 50 million people worldwide and causing approximately 5.3 million deaths annually. According to a systematic review, it is recognized as the major cause of admission to the intensive care unit (ICU) and death worldwide [2,3,4]. In 2017, the World Health Organization (WHO) suggested that sepsis should be recognized as a global health priority, and its prevention, diagnosis, and management should be improved [3,4,5]. Moreover, sepsis is frequently found to be the cause of death due to infections worldwide. Early research predominantly focused on bacterial infection, which underlies the etiology of 80% of adult septic patients [6,7]. In 2020, with the spread of SARS-CoV-2, high mortality, and stressed ICU capacity, research on sepsis turned toward the host response to the invading virus. It has been reported that approximately 2–5% for patients with COVID-19 suffer multi-organ injury after 8–10 days, which is typical of sepsis [8,9,10]. Despite this alarming situation, sepsis, which is little known to the general public, remains relatively neglected.

Billions of dollars have been invested by the government and the pharmaceutical industry in the past 40 years for the development of specific immune system-targeting adjunct therapies for sepsis. However, to date, none of the numerous clinical trials using a single adjuvant treatment has achieved a breakthrough [11,12,13]. Basic and clinical scientists agree that rather than these drugs being ineffective, this observation could be due to the inability to perform continuous immunomonitoring for each patient who may suffer from different conditions and extent of sepsis [14]. Therefore, ongoing research aims to utilize individualized management strategies that are matched to personal clinical profiles [15].

Transglutaminase 2 (TG2) is a multifunctional enzyme that is associated with a variety of physiological and pathological functions, including cell growth, differentiation and development, cell adhesion and morphology, cytoskeletal rearrangement, extracellular matrix stabilization, inflammatory processes, receptor-mediated endocytosis, and apoptosis [16,17]. TG2 is a calcium-dependent transamidase that catalyzes the crosslinking of glutamine to lysine residues within or between proteins [18]. Given these functions, TG2 plays critical roles in the pathogenesis of a variety of human diseases, including inflammatory diseases, such as colitis, celiac disease, rheumatoid arthritis, cardiovascular disease, neurodegenerative diseases, fibrosis, cancer, and sepsis [19,20,21,22,23]. This review aims to provide an overview of the current understanding of the relationship between TG2 and sepsis, as well as updated evidence of the genetic and pharmacological inhibition of TG2 as a potential therapeutic strategy for inflammation and sepsis.

## 2. Role of Inflammation in the Pathophysiology of Sepsis

Sepsis is a syndrome characterized by dysregulated inflammation, microbial and host pattern recognition, tissue hypoperfusion and hypoxia, hypercoagulation, oxidative damage, and immune suppression, which can lead to parenchymal tissue damage and neurological disturbances, resulting in multi-organ failure [24,25,26]. The underlying pathophysiological processes involve the cumulative dysfunction of molecular players, circulating cells including immune cells (macrophages, neutrophils, and lymphocytes), platelets, and endothelium and epithelium [27]. Sepsis, caused by an invading pathogen, is an inflammatory disease that can activate the innate immune response. There are two main paradigms involved in the activation of the innate immune system during sepsis. The first is that intracellular and extracellular danger signals can be simultaneously recognized by complement and germline-encoded pattern recognition receptors (PRRs) on cells, which is critical for the maintenance of immune surveillance. These PRRs sense the presence of pathogen-associated molecular patterns (PAMPs) such as bacterially derived lipopolysaccharides (LPS) and recognize endogenous molecules that reflect cellular damage, termed damage-associated molecular patterns (DAMPs), such as reactive oxygen species (ROS), double-stranded RNA, mitochondrial DNA, and cytochrome C. The cells expressing PRRs include immune cells such as macrophages and dendritic cells (DCs) as well as various nonprofessional immune cells such as epithelial and endothelial populations that are in dynamic equilibrium with the innate immune and coagulation systems [25,26,28,29]. PRR families can be divided into four classes, including transmembrane proteins such as Toll-like receptors (TLRs) and C-type lectin receptors (CLRs), and cytoplasmic proteins such as NOD-like receptors (NLRs) and retinoic acid-inducible gene (RIG)-I-like receptors (RLRs) [28], which can induce complex intracellular signaling. The second process involves the activation of multiple signaling pathways associated with inflammation and cellular metabolism via PAMPs and DAMPs. In other words, the recognition of extracellular danger signals can lead to the recruitment and phosphorylation of pro-inflammatory mediators such as mitogen-activated protein kinases, signal transducers and activators of transcription, and nuclear translocation of nuclear factor-κΒ (NF-κΒ) [25].

Phagocytic cells, including macrophages and neutrophils, play crucial roles in the pathogenesis of sepsis. After stimulation, macrophages release pro-inflammatory mediators such as tumor necrosis factor-α (TNF-α), interleukin-1 (IL-1), IL-6, and IL-8, which not only eliminate the invading bacteria but also cause tissue damage and organ injury [30]. Neutrophils can release regulatory leukotrienes, cytokines, and chemokines to help clear invading pathogens and directly contribute to the elimination of the pathogen by expressing abundant antimicrobial peptides, proteases, and oxidants, and thus, they are the vital components of the innate immune response during sepsis [31,32].

Traditionally, it was thought that at the beginning of sepsis, the body undergoes an overactive inflammatory response, which subsequently develops into a more protracted immunosuppressive phase with the help of anti-inflammatory mediators [33,34]. However, newer paradigms suggest that pro- and anti-inflammatory responses might occur simultaneously in the early stages of sepsis [35]. The immune response initiated by the invading pathogen fails to keep under control excessive inflammation, which is characterized by excessive inflammation and immune suppression. Therefore, patients who survive hospitalization after sepsis often suffer from chronic immune suppression and inflammation [36]. This condition is called persistent inflammatory-immunosuppressive and catabolic syndrome (PICS) [37,38]. PICS involves ongoing inflammation, organ failure, protein catabolism, poor nutrition, poor wound healing, and immunosuppression with increased susceptibility to secondary infections [39]. PICS patients can be identified based on the presence of multiple immunological and physiological defects that occur simultaneously [37,40]. These features facilitate the differentiation of PICS from the “Post-ICU Syndrome”, which leads to a persistent decline in physical, cognitive, and mental health functioning in survivors of ICU hospitalization, regardless of its etiology [41,42]. Some clinical trials have demonstrated that adjunctive immunotherapy with interferon γ (IFN-γ) improves the host immune response in sepsis-induced immunosuppression [43]. However, a recent study demonstrated that IFN-γ produced from invariant natural killer T cells lowered macrophage phagocytosis and promoted post-sepsis immunosuppression [44]. Collectively, these data suggest that sepsis is a complicated and multistep process that is mediated by temporally precise interactions between different cell types and the integration of multiple signals. Therefore, it is important to understand the pathogenesis of sepsis in specific cell types and at different stages of the disease.

## 3. Physiological and Pathological Role of TG in Inflammation

TG is an enzyme that crosslinks lysine and glutamine within or between proteins by forming an isopeptide bond. TG is a family of enzymes consisting of TG1–7 and Factor XIIIa (FXIIIa). TG1, TG3, and TG5 are mainly expressed in the skin and esophagus, where they stabilize the cornified cell envelope by crosslinking the terminally differentiated keratinocytes [45]. TG1 is essential for the assembly and organization of the barrier structures to form a cornified envelope [46]. TG1-deficient mice show the ichthyosiform skin phenotype and develop massive hyperkeratosis [47]. TG3-deficient mouse skin is more responsive to the action of the pro-inflammatory drug imiquimod than WT mouse skin, resulting in psoriasis [48]. TG5 contributes to the hyperkeratotic phenotype in ichthyosis and psoriasis [49]. TG2 is ubiquitously expressed and is regulated by Ca^2+^ and guanosine triphosphate (GTP). TG4 is specifically expressed in the prostate and crosslinks proteins in the seminal vesicle fluid [50]. FXIIIa is a blood coagulation factor that forms fibrin-based clots by crosslinking [51].

**Figure 1:**
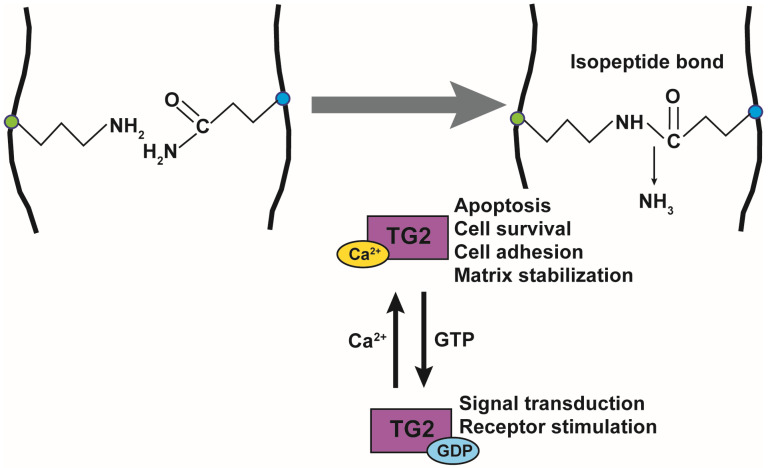
Transglutaminase 2 activity and function. Transamidation activity of transglutaminase 2 (TG2) is activated by Ca^2+^ and catalyzes the formation of an isopeptide bond. TG2 function is also regulated by guanosine triphosphate (GTP) binding. Ca2+-binding to TG2 regulates apoptosis, cell survival, cell adhesion, and matrix stabilization, while the binding of GDP to TG2 regulates signal transduction and receptor activation.

The function of TG2 has been extensively studied and is known to be regulated by Ca^2+^ and GTP. TG2 is composed of 667 amino acids and has a catalytic core domain with an activity center similar to cysteine proteases. The GTP-bound TG2 exists in a closed form and its conformation changes to the open form upon Ca^2+^-binding, which also activates transamidase activity (Figure 1). Its transamidation activity can be blocked by using Ca^2+^ chelators such as EDTA and EGTA, inhibiting cysteine in the catalytic core region using cystamine (CTM), and mimicking the substrate glutamine using a 6-Diazo-5-Oxo-L-Norleucine (DON). TG2 is active in the extracellular matrix, plasma membrane, cytoplasm, mitochondria, recycling endosomes, and nucleus, and it crosslinks various substrate proteins [52]. TG2 plays an important role in apoptosis, cell survival, cytoskeletal function, cell motility, adhesion, and signal transduction. The opposing roles of TG2 have been reported in the brain, liver, cancer, pancreas, and angiogenesis [53].

**Figure 2:**
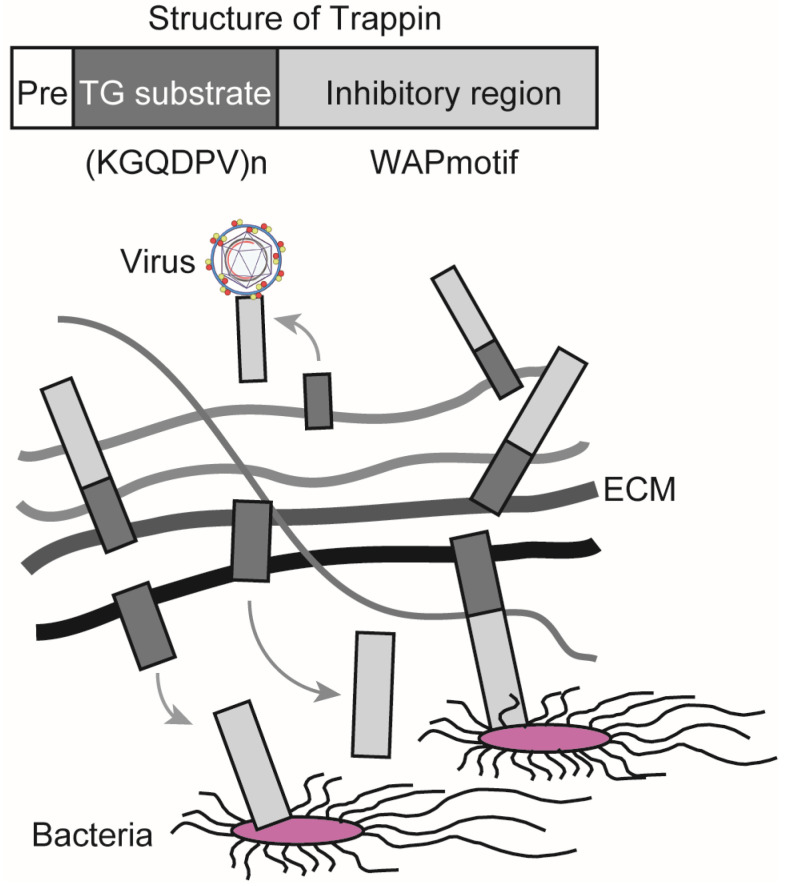
Trappin is responsible for antibacterial and antiviral activities. Trappin has a signal sequence for secretion (Pre), a TG substrate domain with a repeat sequence KGQDPV, and an inhibitory region that has a whey acidic protein (WAP) motif. Trappin is crosslinked to the extracellular matrix (ECM) by TG, whereby an inhibitory region is released into the extracellular space. The WAP motif exerts antibacterial and antiviral activities.

TG exerts antibacterial and antiviral effects through its substrates. Elafin is a member of the Trappin family and has been reported to suppress the growth of *Pseudomonas aeruginosa,* infection and replication of human immunodeficiency virus 1 (HIV-1), and infection with herpes simplex virus 2 (HSV-2) [54,55,56]. Trappin has a signal sequence for secretion (Pre), a TG substrate domain, and an inhibitory region containing a whey acidic protein (WAP)-motif that shows antibacterial and antiviral activities. Elafin is expressed in the skin, trachea, and vagina, and it is efficiently crosslinked to the extracellular matrix (ECM) through the TG substrate domain, which consists of a repeating sequence with lysine (K) and glutamine (Q)-rich “KGQDPV” sequence. TG controls the localization of Trappin by covalent crosslinking, releasing the WAP-motif from ECM, which, in turn, increases its antibacterial and antiviral activities (Figure 2) [57]. Women demonstrating resistance to HIV-1 infection have been shown to have a higher expression of Elafin with only the 6 kDa WAP motif in the reproductive organs [56]. Recombinant Elafin and Trappin-2 inhibit HIV-1 replication in T cells [57,58]. The Trappin family was formed through gene duplication, especially in porcine relatives, and underwent accelerated evolution [59,60]. They are thought to function as part of the innate immune response to various bacteria and viruses.

Our previous study showed that both the transaminase activity and expression of TG2 were increased in the livers of LPS-induced sepsis mice [22]. Single-cell RNA sequencing (scRNA-seq) provided a comprehensive view of TG2 gene expression in liver-composing cells [61]. Data mining of the scRNA-seq database and immunofluorescence staining revealed that the expression of TG2 in the liver under steady-state conditions was mainly detected in macrovascular and sinusoidal endothelial cells [22,61,62]. However, under inflammatory conditions, enhanced TG2 expression was observed in the F4/80-positive midzonal macrophages, suggesting that activated macrophages were the major cellular source of TG2 in the livers of sepsis mice [22]. The increase in TG2 gene expression in macrophages is believed to be involved in the activation cycle of the inflammatory process [19]. The stimulation of Toll-like receptor 4 (TLR4) by LPS induces the release of critical pro-inflammatory cytokines such as tumor necrosis factor-alpha (TNF-α), which triggers a series of intracellular events that result in the activation of transcription factor nuclear factor-kappa B (NF-κB) and the activation of mitogen-activated protein kinases (MAPKs) [63,64]. The gene expression of TG2 is directly regulated by NF-κB through the NF-κB-binding motif in its promoter region [65,66]. In contrast, TG2 is also critical in NF-κB activation by promoting thrombin-induced DNA binding and serine phosphorylation of RelA/p65 [67] or by stimulating the polymerization of the inhibitory subunit α of NF-κB (I-κBα) [19,68]. The TG2-dependent activation of NF-κB might further promote the survival of macrophages [69] and a continuous activation cycle during the inflammatory process in sepsis.

The dysregulation of apoptosis in immune and nonimmune cells plays a critical role in the pathogenesis of sepsis [70,71]. Opposing roles of TG2 have been reported in the regulation of cell death and apoptosis [53]. TG2 gene expression in peripheral blood mononuclear cells and lymph nodes was induced following human immunodeficiency virus (HIV) infection [72]. An increased transaminase activity of TG2 was observed in lymphocytes undergoing apoptosis during the process of HIV infection [73]. Our group has shown that TG2 has a nuclear localization signal (NLS) and a nuclear export signal (NES). Its localization to the nucleus depends on importin, and its export from the nucleus depends on chromosomal region maintenance 1 (CRM1) [74]. TG2 was shown to translocate into the nucleus, and there was a crosslink that inactivated the transcription factor Sp1, inducing apoptosis by suppressing c-Met expression [75]. Following the co-culture of *Candida albicans* and hepatocytes, TG2 translocates into the nucleus and is activated by ROS generated by *Candida albicans* [76]. The induction of TG2 was also involved in the cell death induced by retinoids and fatty acids in hepatic cells [77,78,79]. In the LPS-induced sepsis mouse model, TG2 in the hepatic macrophages was activated, and the inhibition of TG2 activation prevented LPS-induced hepatic damage, as indicated by the serum level of ALT, which is a well-established indicator of hepatocyte cell death [22]. In contrast, the anti-apoptotic role of TG2 as a G protein has also been reported [80]. In TNF-α-induced sepsis mice, TG2 inhibited liver injury by downregulating TNF-α-induced expression of pro-death proteases caspase 3 and cathepsin D [81]. Thus, TG2 appears to be involved in host–pathogen interactions and plays multiple roles in the mechanisms of cell response to inflammatory substances in a context-dependent manner.

Celiac disease is a type of autoimmune disease that occurs primarily in the intestine, causing inflammatory disorders and villous atrophy triggered by gluten intake. Celiac disease affects approximately 1% of people in Europe and the United States [82]. It has been reported that antibodies against gliadin and TG2 are detected in patients with celiac disease [83,84]. In addition, Gln (Q) of gliadin is converted to Glu (E) by the deamination activity of TG2. This is thought to increase antigenicity, activate the immune system through the activation of T and B cells, and exacerbate autoimmune disease [85].

It has been reported that mucosal permeability increases in celiac disease because it causes inflammatory disorders in the small intestine [86], and that bacteria are more likely to bind to the small intestinal epithelium of celiac disease patients [87]. It has been suggested that the risk of causing sepsis is increased. Using Swedish national health resisters, Ludvigsson et al. showed that patients with celiac disease are more likely to develop sepsis, especially caused by pneumococcal and staphylococci, based on patient information that is not a celiac disease or celiac disease [88]. Thus, sepsis is also associated with celiac disease, which is exacerbated by the deamination of gliadin by TG2. This suggests that suppression by a TG2-specific inhibitor works effectively to treat celiac disease. However, a TG2-specific inhibitor could induce side effects because of the possible increased membrane permeability of the gut.

## 4. Targeting TG2 in Inflammation and Sepsis: Evidence from Knockout Mouse Models

Targeted mutant mice, such as those with a gene knockout (KO), play a vital role in understanding the integrated physiological and pathological functions of a protein in a tissue-specific context [89]. Three TG2 KO mouse lines [90,91,92] were generated using genomic DNA from the 129 sub-strain to target the catalytic domain of the *Tgm2* gene. These genetically engineered mouse models have been used to explore the biological roles of TG2 in diverse pathophysiological contexts (reviewed in [93]). Under physiological conditions, TG2 KO mice were viable, with no obvious phenotypic or developmental abnormality compared to wild-type (WT) mice [90,91,92]. This could be partly explained by the compensatory activation, either transcriptional upregulation or upregulation of transamidation activity, of other TGs [94,95]. Under pathological conditions, a reduced inflammatory response has been reported in TG2 KO mice. In the pulmonary fibrosis [96] and allergic asthma model [97], reduced inflammation was observed in the TG2 KO mice compared to WT mice, which was possibly due to the regulation of T helper cells and the recruitment of both innate and adaptive immune cells. In a Parkinson’s disease (PD) model, the TG2 KO mice reversed the behavioral manifestations of PD by downregulating the release of inflammatory mediators such as histamine, leukotrienes, and cytokines by mast cells in the substantia nigra, suggesting that TG2 might contribute to neuroinflammation and neurodegeneration [98]. In a multiple sclerosis (MS) model, TG2 was present in the major histocompatibility complex class II-positive infiltrating cells in active MS lesions [99]. TG2 KO mice showed reduced experimental autoimmune encephalomyelitis, supporting a role of TG2 in MS pathogenesis [97,99]. The underlying mechanism was related with the inhibition of influx of immunomodulatory macrophages into the central nervous system [99]. In the UV irradiation-induced skin inflammation model, the TG2 KO mice showed reduced pro-inflammatory cytokine production in the keratinocytes [100]. Mechanistically, UV irradiation induced the activation of phospholipase C and calcium release from the endoplasmic reticulum, which leads to TG2 activation but does not induce TG2 gene expression [100]. On the other hand, an anti-inflammatory effect was also reported in TG2 KO mice. In a peritonitis model, TG2 KO mice showed hyperinflammation after exposure to monosodium urate (MSU) crystals [101]. In vitro mechanistic analysis revealed that TG2 overexpression suppressed MSU crystal-induced IL-1β and TNF-α production in macrophages through a transforming growth factor-β (TGF-β)-dependent pathway [101].

The role of TG2 deficiency in the pathology of sepsis is controversial. Following LPS-induced inflammation, a decrease in NF-κΒ activation and the sequestration of polymorphonuclear leukocytes (PMNs) was observed in the lungs of TG2 KO mice, suggesting that TG2 promotes endothelial cell inflammation and lung PMN infiltration [67]. In TNF-α-dependent septic mice, TG2 exhibited a protective role in liver injury, and TG2 KO mice showed increased liver damage compared to WT mice [81]. However, it has also been reported that TG2 KO mice improve survival after LPS-induced septic shock compared to WT mice. A marked reduction in the inflammatory response and attenuated organ damage, partly through the regulation of DC differentiation and function, was observed in the TG2 KO mice [19,21]. One explanation for these inconsistencies is that targeted mutations might have different phenotypes in different strain backgrounds, which, in combination with the difference in study design, significantly influences the reliability and reproducibility of the studies. Another explanation is that it is likely that the effect of TG2 on the progression of inflammation and sepsis may depend on the targets present in different cells and tissues, such as the apoptotic stress in epithelial cells and inflammatory stress in immune cells. Indeed, a dual role for TG2 has been reported to promote and protect the pathology of liver injury and obesity-related inflammation [62,75,102,103].

Given the critical roles of genetic knockout animal models in exploring the function of TG2 in the impairment of cell response in the presence of sepsis inducers, further studies using genetically engineered mouse models with tissue-specific deletion of *Tgm2* are required to explore the cell type and disease stage-dependent roles of TG2 in inflammation and sepsis. As proof of principle, endothelial cell-specific deletion of TG2 provided direct evidence demonstrating TG2 controls allergic inflammation by regulating the recruitment of eosinophils into the lung endothelium [104].

## 5. Targeting TG2 in Inflammation and Sepsis: Evidence from Pharmacological Inhibition

**Figure 3:**
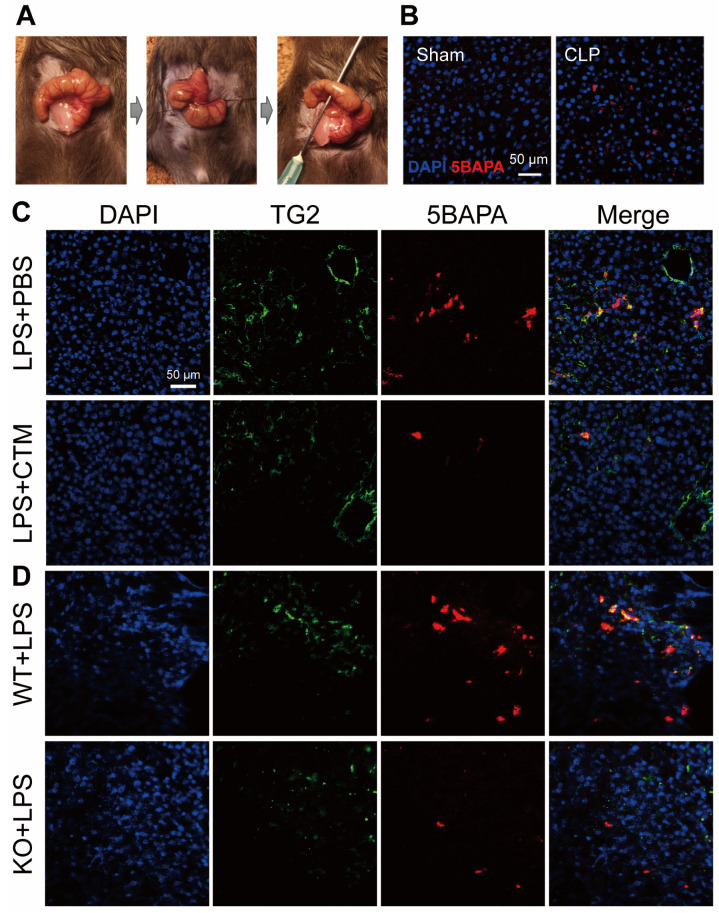
TG2 activity in the liver of septic mice. (**A**) Representative images from the cecal ligation and puncture (CLP) procedures. (**B**) TG2 activity in the liver of CLP-operated mice. Immunofluorescence staining with 4′,6-diamidino-2-phenylindole (DAPI) (blue) and 5-biotinamidopentylamine (5BAPA) (red) in frozen liver sections obtained from mice 48 h after sham or CLP operations. Scale bar: 50 μm. (**C**) TG2 activity in the liver of lipopolysaccharides (LPS)-injected mice. Immunofluorescence staining with DAPI (blue), TG2 (green), and 5BAPA (red) in frozen liver sections obtained from mice injected intraperitoneally with LPS in the presence and absence of CTM. Scale bar: 50 μm. (**D**) TG2 activity in the liver of LPS-injected TG2 knockout (KO) mice. Immunofluorescence staining of DAPI (blue), TG2 (green), and 5BAPA (red) in frozen liver sections obtained from mice injected intraperitoneally with 10 mg/kg LPS for 24 h. Scale bar: 50 μm. These images have been adapted from a previous study [22].

Given the critical role of TG2 in a wide range of physiological disorders, the development of TG2 inhibitors has been the subject of intense research. CTM is a symmetric disulfide compound and is one of the earliest known pharmacological inhibitors of TG2, acting via competitive amine inhibition [105] and irreversible oxidation [106]. There is abundant evidence indicating that the suppression of TG2 transamidation with CTM has a beneficial effect against inflammation. CTM significantly delayed neuroinflammation in amyotrophic lateral sclerosis by inhibiting the TG2-induced oligomerization of superoxide dismutase 1 [107]. In a rat model of inflammatory bowel disease, CTM reduced the severity of colitis, which was associated with a decrease in TG2 activity and the production of pro-inflammatory cytokines such as TNF-α and IL-6 [108]. During lung inflammation in cystic fibrosis (CF), CTM restored TG2-mediated crosslinking of beclin 1 and subsequently rescued defective autophagy, aggresome formation, and the CF airway phenotype [109]. In contrast to its well-known anti-inflammatory effect, little is known about the effect of CTM on sepsis. Recently, we established an ex vivo imaging system to detect the in vivo transamidation activity of TG2 based on the incorporation of a biotinylated substrate for TG2, 5-biotinamidopentylamine (5BAPA), in the liver of LPS and cecal ligation and puncture (CLP)-induced sepsis mouse model (Figure 3A,B) [22]. We found that an LPS challenge and CLP operation dramatically induced the expression and activation of TG2 in the midzonal F4/80/CD80-positive M1 macrophages in the murine liver. The administration of CTM, 30 min before the LPS challenge, almost completely suppressed the 5BAPA signals in the liver and ameliorated the LPS-induced liver injury, suggesting that targeting TG2 with CTM holds great promise as a therapeutic strategy for sepsis (Figure 3C,D) [22]. Interestingly, pharmacological inhibition of TG2 with CTM at an early stage and not the late stage alleviated *Schistosoma japonicum*-induced liver fibrosis [110]. In addition to their therapeutic impact, pharmacological inhibitors such as CTM could be used as mechanistic probes to explore the disease stage-dependent physiological and pathological roles of TG2.

However, it should be noted that the inhibitory effect of CTM on transamidation activity is not specific to TG2 [111]. In addition, CTM can interact with other signaling pathways involved in the regulation of cell proliferation and death, such as the caspase signaling pathway [112]. Therefore, recent research has focused on the development of selective inhibitors against TG2 for their therapeutic potential (reviewed in [113]). These novel irreversible inhibitors of TG2, such as NC9 and VA4 [114], which specifically react with Cys-277 in the TG2 transamidation site and inhibit both transamidation and GTP-binding activities, are potent anticancer agents that inhibit the survival of cancer stem cells [115]. Very recently, an in vitro study showed that NC9 effectively reduced the levels of pro-inflammatory cytokines such as monocyte chemotactic protein 1, IL-1β, and TNF-α, in combined all-trans retinoic acid and arsenic trioxide-treated acute promyelocytic leukemia cells [116]. Evidence for genetic and pharmacological inhibition of TG2 during inflammation and sepsis is summarized in Table 1. Further animal studies are required to explore the therapeutic efficacy and safety of these novel specific inhibitors of TG2 in both inflammation and sepsis.

Since TG2 is dramatically expressed in the livers of septic mice; it could be used as a marker of sepsis. Although TG2 has opposing roles in inflammation, it has been suggested that TG2 exacerbates sepsis, fibrosis, and neurodegenerative diseases. Therefore, TG2 inhibitors, such as CTM, could be effective therapeutic agents. Further translational research is expected to evaluate the clinical relevance of TG2-specific inhibitors in the treatment of sepsis in the future.

## Figures and Tables

**Table 1:** Evidence for genetic and pharmacological inhibition of TG2 during inflammation and sepsis.

	Experimental Evidence	Mechanistic Insight	Role of TG2	Reference
**TG2 KO mice**				
	TG2 KO protects from LPS-induced septic shock and mortality	TG2 promotes NF-κΒ activation and DC differentiation	⇧ Promoting	[19] [21]
	TG2 KO reduces LPS-induced EC inflammation and lung PMN infiltration	TG2 promotes DNA-binding and transcriptional activity of RelA/p65 and NF-κB activation	⇧ Promoting	[67]
	TG2 KO exacerbates TNF-α-dependent septic shock and liver injury	TG2 inhibits TNF-α-induced expression of caspase 3 and cathepsin D	⇩ Protective	[81]
	TG2 KO reduces inflammation and fibrosis after noninfectious pulmonary injury	TG2 induces the secretion of IL-6 in epithelial cells but not inflammatory cells and contributes to the effector phase of fibrogenesis under the control of TGF-β in fibroblasts	⇧ Promoting	[96]
	TG2 KO ameliorates experimental autoimmune encephalomyelitis, which is an autoimmune disease model for multiple sclerosis	TG2 promotes differentiation of CD4(+) T cells into IL-17- or IFN-γ-producing cells andmacrophage migration into the central nervous system associated with the induction of RhoA GTPase activity, and iNOS and TNF-α production	⇧ Promoting	[97] [99]
	TG2 KO decreasesneuroinflammation in MPTP-induced Parkinson’s disease model	TG2 promotesthe release of inflammatory mediators such as histamine, leukotrienes, and cytokines by mast cells in the substantia nigra	⇧ Promoting	[98]
	TG2 KO reduces pro-inflammatory cytokine production in UV-irradiated keratinocytes	UV irradiation stimulates TG2 activity through phospholipase C-dependent endoplasmic reticulum calcium release	⇧ Promoting	[100]
	TG2 KO increases hyper inflammatory responses in a peritonitis model	TG2 inhibits MSU crystal-induced IL-1β and TNF-α production in macrophages through a TGF-β-dependent pathway	⇩ Protective	[101]
**TG2 inhibitors**				
**Cystamine**	Cystamine inhibits LPS-induced liver injury	TG2 is mainly expressed and activated in midzonal F4/80/CD80+ M1 macrophages in the livers of septic mice	⇧ Promoting	[22]
	Cystamine inhibits neuroinflammation in amyotrophic lateral sclerosis	TG2 catalyzes the oligomerization of superoxide dismutase 1 and induces TNF-α, IL-1β, and nitric oxide in microglial cells	⇧ Promoting	[107]
	Cystamine ameliorates TNBS-induced colitis in a rat model of inflammatory bowel disease	TG2 activity is associated with the production of mucosal TNF-α and serological IL-6	⇧ Promoting	[108]
	Cystamine rescues defective CFTR-induced cystic fibrosis	TG2 catalyzes the crosslinking of beclin 1, leading to the sequestration of phosphatidylinositol-3-kinase complex III, accumulation of p62, and aggresome formation	⇧ Promoting	[109]
**NC9**	NC9 reduces pro-inflammatory cytokine production in combined ATRA and ATO-treated APL cells	TG2 leads to inflammation, which is probably due to reactive oxygen species production	⇧ Promoting	[116]

⇧, promoting role in inflammation or sepsis; ⇩, protective role in inflammation or sepsis; APL, acute promyelocytic leukemia; ATO, arsenic trioxide; ATRA, all-*trans* retinoic acid; CFTR, cystic fibrosis transmembrane conductance regulator; DC, dendritic cell; EC, endothelial cell; IFN-γ, interferon-γ; IL-1β, interleukin-1β; IL-17, interleukin-17; iNOS, inducible nitric oxide synthase; KO, knockout; LPS, lipopolysaccharide; MPTP, 1-methyl-4-phenyl-1,2,3,6-tetrahydropyridine; MSU, monosodium urate; NF-κΒ, nuclear factor-κΒ; PMN, polymorphonuclear leukocyte; TGF-β, transforming growth factor-β; TNBS, 2,4,6-trinitrobenzene sulfonic acid; UV, ultraviolet.

## Data Availability

Data sharing not applicable. No new data were created or analyzed in this study.
